# Chrysophanol Relieves Cisplatin-Induced Nephrotoxicity *via* Concomitant Inhibition of Oxidative Stress, Apoptosis, and Inflammation

**DOI:** 10.3389/fphys.2021.706359

**Published:** 2021-09-29

**Authors:** Siqing Ma, Heng Xu, Weihua Huang, Yongchao Gao, Honghao Zhou, Xiong Li, Wei Zhang

**Affiliations:** ^1^Department of Clinical Pharmacology, Xiangya Hospital, Central South University, Changsha, China; ^2^Hunan Key Laboratory of Pharmacogenetics. Institute of Clinical Pharmacology, Central South University, Changsha, China; ^3^Engineering Research Center of Applied Technology of Pharmacogenomics, Ministry of Education, Changsha, China; ^4^National Clinical Research Center for Geriatric Disorders, Changsha, China; ^5^Department of Laboratory Medicine, State Key Laboratory of Biotherapy and Cancer Center, West China Hospital, Sichuan University, Chengdu, China; ^6^The First Affiliated Hospital of Guangdong Pharmaceutical University, Guangdong, China

**Keywords:** chrysophanol, cisplatin, acute kidney injury, oxidative stress, apoptosis, inflammation

## Abstract

Cisplatin (CDDP) is one of the most frequently prescribed chemotherapy medications. However, its nephrotoxicity which often leads to acute kidney injury (AKI), greatly limits its clinical application. Chrysophanol (CHR), a mainly active anthraquinone ingredient, possesses various biological and pharmacological activities. In this study, we aimed to investigate the underlying protective mechanisms of CHR against CDDP-induced AKI (CDDP-AKI) using C57BL/6 mouse and human proximal tubule epithelial cells. *In vivo*, we found that pre-treatment with CHR greatly relieved CDDP-AKI and improved the kidney function and morphology. The mechanistic studies indicated that it might alleviate CDDP-AKI by inhibiting oxidative stress, apoptosis, and IKKβ/IκBα/p65/transcription factor nuclear kappa B (NF-κB) inflammation signaling pathway induced by CDDP. Moreover, we found that the cell viability of HK2 cells reduced by CDDP was partially rescued by CHR pre-incubation. Flow cytometry results further indicated that CHR pre-incubation suppressed CDDP induced cellular reactive oxygen species (ROS) generation and inhibited cell apoptosis in a dose-dependent manner. In summary, our results suggested that CHR might be a novel therapy for CDDP-induced AKI.

## Introduction

Cisplatin, cisplatinum, or cis-diamminedichloroplatinum II (CDDP), as a member of platinum agents, is widely used in the treatment of various tumors, such as sarcomas, solid carcinomas (e.g., small cell lung cancer, and ovarian cancer), lymphomas, and germ cell tumors (Yao et al., 2007; Manohar and Leung, 2018). However, CDDP application is largely restricted by its most well-known complication, acute kidney injury (AKI; Bellomo et al., 2012). In clinic, approximately one-third of patients that received CDDP-based chemotherapy will develop AKI (Shord et al., 2006). While volume expansion and magnesium supplementation have been performed to prevent CDDP induced AKI (CDDP-AKI), the outcome remains unsatisfactory. Other potential approaches including antioxidants and blockers of cisplatin transport are also explored, but still far from satisfaction, due to their limited efficacy. Therefore, there is an urgent need to identify new agents to overcoming CDDP-AKI.

As is known, CDDP affects various signal transduction pathways and ultimately leads to renal tubular injury, interstitial inflammatory reaction, and vascular damage (Ozkok and Edelstein, 2014). It was reported that DNA damage, both intrinsic and extrinsic apoptosis, inflammation, and oxidative stress were activated in the development of CDDP-AKI. Among them, massive cell death as well as devastating inflammatory pathologies are what directly lead to the loss of organ function. Interestingly, proximal tubule epithelial cells are highly sensitive to apoptosis and cell damage at this location contributes most to organ failure (Townsend et al., 2003). CDDP can activate both the BCL2-regulated mitochondrial dysfunction (also called intrinsic apoptotic pathways) and the extrinsic death receptor pathway *via* tumor necrosis factor alpha (TNF-α) production (Havasi and Borkan, 2011). It can also trigger apoptosis by activating p53 pathway (Wei et al., 2007). Besides, various pro-inflammatory cytokines, including Interleukin 1 beta (IL-1β), IL-6, or TNF-α and infiltrating inflammatory cells are demonstrated to participate in the CDDP-AKI. At this point, the transcription factor nuclear kappa B (NF-κB) signaling pathway, especially the canonical one characterized by activated IKKβ and phosphorylated IĸBα, dynamically regulates the inflammatory response to CDDP stimulation (Ozkok et al., 2016).

Following cellular uptake of CDDP, the increase in reactive oxygen species (ROS) levels appears in the early stages of the process and is mainly confined to the tubules without capillary blood flow, which increases tissue oxidative stress and promotes apoptosis as well as activates inflammatory cytokines in the kidney (Santos et al., 2007; Chirino and Pedraza-Chaverri, 2009). According to current studies, ROS can be generated by NADPH oxidases of the NOX family, which stimulates the transfer of electrons from NADPH to molecular oxygen, thereby generating ROS (Bedard and Krause, 2007). NOX4 and NOX2 are the main form in the kidney and they can act as oxygen sensors and regulate the synthesis of erythropoietin in the renal cortex (Gill and Wilcox, 2006; Holterman et al., 2015; Thallas-Bonke et al., 2015). Until now, numerous kidney diseases, including AKI, diabetic nephropathy (DN), and chronic kidney disease (CKD) are proved to be associated to the abnormal of NOX2/4 expression and ROS level (Babelova et al., 2012; Gorin and Block, 2013; Thallas-Bonke et al., 2015; Meng et al., 2018). High expression of NOX4 plays an important role in kidney damage by affecting the level of oxidative stress. In ischemic/reperfusion (I/R) AKI, it can interact with Toll Like Receptor 4 (TLR4) and stimulate the production of ROS (Qi et al., 2015).

Based on these molecular mechanisms, numerous studies tried to excavate promising potential therapeutic agents. Among them, N-acetylcysteine (NAC), quercetin, curcumin, and arjunolic acid were some well-studied nephroprotective drugs (Casanova et al., 2020). Surprisingly, none of these products has yet stepped to clinical studies for the prevention of CDDP-AKI. As an example, NAC prevents both the death receptor and the mitochondrial apoptotic pathways induced by CDDP in preclinical studies (Wu et al., 2005). At the clinical level, however, the use of NAC was not effective in attenuating the toxicity of CDDP in patients with head and neck cancer (Visacri et al., 2019). This means that exploration of alternative candidates is still urgently needed.

Chrysophanol (CHR, also known as chrysophanic acid and 1,8 dihydroxy-3-methyl-anthraquinone) is an anthraquinone component extracted from various herbs, such as Rheum palmatum L, Polygonum multiflorum, and Cassia obtusifolia. Rheum palmatum L was a traditional Chinese medicine used for centuries in different fields, including pharmaceutical, health care, and cosmetics (Yusuf et al., 2019). Multiple studies showed that it exerted a wide range of beneficial effects including tumor-suppression, virucidal activity, neuroprotection, antiplatelet and anticoagulant, protection from diabetes, inflammatory responses, hepatic, and pulmonary injury (Xie et al., 2019). The molecular mechanisms and pathways underlying its cardiovascular, nervous system, and liver protective effects may be linked to its MAPK modulation (subsequent pro-inflammatory mediator inhibition) or ROS generation inhibition (subsequent mitochondrial proapoptotic factors induction; Lin et al., 2015; Chae et al., 2017). Strikingly, a meta-analysis found that *Rheum officinale* had a beneficial effect on renal function (Cao et al., 2017). Further to this, another study suggested the protective potential of Rheum turkestanicum against CDDP nephrotoxicity *via* oxidative stress reduction (Hosseini et al., 2018). However, there is very limited evidence for CHR’s protective effects against kidney damage. Whether CHR can relieve CDDP-AKI has also not been evaluated. In our study, we aimed to investigate the protective role and potential mechanisms of CHR against CDDP-AKI *in vitro* and *in vivo*.

## Materials and Methods

### Reagents

Chrysophanic Acid (CHR) and CDDP were purchased from Sellect (Houston, TX, United States). Dulbecco’s modified Eagle medium F12 (DMEM/F12), Trypsin-EDTA (0.25%), and Antibiotic-Antimycotic solution were purchased from Gibco (United States). Fetal bovine serum (FBS) was supplied by CellMax (Beijing, China). Other consumable materials for cell culture were acquired from Greiner Bio-One (Essen, Germany). F4/80 (GB11027; Servicebio, Wuhan, China), c-caspase3 (GB11532; Servicebio, Wuhan, China), and Lipocalin-2/neutrophil gelatinase-associated lipocalin (NGAL; ab125075; abcam, Shanghai, China) antibody were used for immunohistochemistry (IHC). Antibody of Lipocalin-2/NGAL (NBP1-45682) was provided by Novus Biologicals (Littleton, United States). Antibodies of Bax (50599-2-Ig,) and Bcl2 (26593-1-AP) were provided by Proteintech (Wuhan, China). Antibodies of cleaved Caspase 3 (cst9664), NF-κB p65 (cst6956), Phospho-NF-κB p65 (cst 3033), IκBα (cst4814), phospho-IκBα (cst9246), IKKβ (cst2678), phospho-IKKα/β (cst2697), and GAPDH (cst2118) were provided by cell signaling technology Inc. (MSA, United States).

### Animals

Animal experiments were performed according to the protocol approved by the Institutional Animal Care and Use Committee of Central South University. Specific pathogen-free (SPF) male C57BL6/J mice (aged 8weeks, weighted 20–25g) were purchased from Hunan SJA Laboratory Animal Co., Ltd. (Certificate: SCXK2019-0004; Approval number: 2018sydw0154). All experimental mice were maintained in the SPF barrier facility of Department of laboratory Animals in Central South University for 7days before being used in the experiments and were fed with standard laboratory food and water.

### Animal Model Establishment

**Figure 1:**
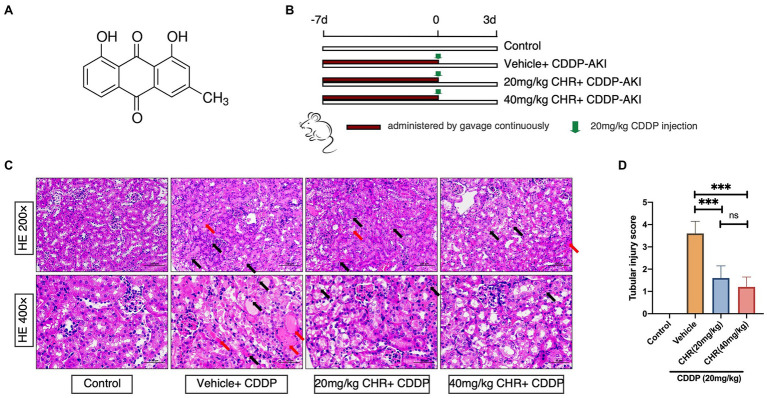
Chrysophanol (CHR) pretreatment attenuated cisplatin (CDDP)-induced kidney morphological damage. C57 mice were administered intragastrically with 20 and 40mg/kg/day for 7days followed by 20mg/kg CDDP intraperitoneal injection or the same volume of vehicle. **(A)** Structural formula of CHR (1,8-dihy-droxy-3-methylanthraquinone). **(B)** Schematic diagram showing the animal experimental design. **(C)** Representative hematoxylin-eosin (HE)-stained sections of the left kidney. Upper panel: 200 ×, Scale bar, 100μm; lower panel: 400 ×, Scale bar, 50μm. Black arrows indicate epithelial cells with apoptotic nuclear morphology (hyperchromatic pleomorphic or fragmental nuclei). Red arrows indicate kidney tubular casts. **(D)** Quantitative analysis of tubular injury with HE stains. ^***^p<0.001 vs. the CDDP group, *n*=7. ns, no statistical difference.

A total of 28 male C57BL6/J mice (aged 8weeks) were chosen and randomly divided into four groups. For each group, mice were treated with vehicle (control group), CDDP only, CDDP and 20mg/kg CHR, and CDDP and 40mg/kg CHR. CHR was administrated by gavage for 7 days (Figure 1A). The mouse model of CDDP-AKI was induced by a single intraperitoneal injection of 20mg/kg CDDP. All mice were sacrificed after 72h with CDDP administration. Kidneys were collected for further analysis after perfusion with saline.

### Renal Function Measurement

Renal function was represented by the levels of serum creatinine (SCr) and blood urea nitrogen (BUN) using an enzymatic assay performed by laboratory department of Xiangya Hospital (Changsha, China).

### Oxidative Stress Evaluation

Plasma superoxide dismutase (SOD) and glutathione peroxidase (GPx) levels were detected by enzymatic colorimetric based SOD and GPx assay kits (Nanjing Jiancheng Bioengineering Institute, Nanjing, China). All procedures were performed following the manufacturer’s instructions.

### Cytokine Level Measurement

IL-1β, IL-6, and TNF-α levels in the serum were measured using ELISA for quantitative detection of mouse IL-1 beta (catalogue number 88-7013), IL-6 (88-7064), and TNF-alpha (88-7324) purchased from Invitrogen (Thermo Fisher Scientific), according to the manufacturers’ instructions.

### Histopathological Examination

Mouse kidney tissue samples were first fixed in 4% paraformaldehyde for over 24h and then embedded in paraffin. Paraffinized sections were cut into 4μm slices and performed hematoxylin-eosin (HE) staining or IHC. Kidney injury degree was determined by semiquantitative grading of tubular necrosis, cast formation, and tubular dilation on a scale from 0 to 4 as follows: 0=normal; 1=injury <20%; 2=20–50%; 3=50–80%; and 4=>80%. For each slide per mouse, 10 areas were selected for grading using low-power fields (magnification 20x). Immunohistochemical staining for NGAL and F4/80 was conducted as described previously. All sections were imaged by NIKON Eclipse Ci microscope (NIKON digital sight DS-FI2, made in JAPAN) and quantified with Image J (NIH Image J system version 1.53, Bethesda, MD). The semi-quantification of IHC staining in kidney tissue sections was analyzed by ImageJ Fiji software (version Fiji for Mac OS X; WS Rasband, National Institute of Health, Bethesda, MD). At least eight random areas of tissues of each group were selected for the mean Gray Value analysis following the standard recommended measurement protocol (Crowe and Yue, 2019).

### Cell Culture and Treatment

Proximal tubular cell line human kidney 2 (HK2) was obtained from the National Collection of Authenticated Cell Cultures (Catalogue number: SCSP-511, Shanghai, China) and routinely cultured in DMEM-F12 medium containing 10% FBS/1% antibiotics at 37°C, 5% CO_2_. All cells were washed with PBS and trypsinized with 0.05% (w/v) Trypsin- 0.53mM EDTA solution for passaging. For CDDP-induced AKI cell model, HK2 cells were seeded in 6-well or 96-well plates and treated with 20μM cisplatin for 48h. HK2 cells were pretreated with vehicle control, 5 or 20μM CHR for 24h before CDDP stimulation.

### Cell Viability Assay

Cell viability was assessed using a Cell Counting Kit-8 (CCK8) assay. HK2 cells were planted into a 96-well plate with the concentration of 5,000 cells per well. After applied treatment for each group and further incubation, 10μl WST-8 dye per well was added for 1h according to the manufacturer’s instruction. The optical density (OD) absorbance was detected by at the wavelength of 450nm.

### Lactate Dehydrogenase Assay

CDDP induced killing and CHR mediated protection of susceptible HK2 cell line was determined using lactate dehydrogenase (LDH) release (Beyotime Biotech) as output for cell lysis. The further calculation of LDH release percentage was also conducted with reference to the manufacturer’s instructions as maximum LDH release activity minus spontaneous LDH release activity.

### Quantitative Real-Time PCR

Kidney tissues and hk2 cells were harvested for total RNA extraction by RNAiso Plus (Takara). Reverse transcription was carried out by PrimeScript™ RT reagent Kit with gDNA Eraser. TB Green® Premix Ex Taq™ (Tli RNaseH Plus) was applied for qPCR reactions performed by LightCycler 480 instrument (Roche Applied Science). All reagents mentioned in this part were purchased from Takara Biomedical Technology (Beijing). Relative mRNA expression data were analyzed by 2-ΔΔCt method and the corresponding primer sequences were described in Supplementary Table 1.

### Flow Cytometry Assay

Both apoptosis and intracellular ROS generation detection of HK2 cells were measured *via* flow cytometry. Flow cytometry analysis of apoptosis in HK2 cells caused by exposure to CDDP and CHR was carried out using the Annexin V-FITC Apoptosis Detection Kit (Invitrogen, 88–8005) according to the manufacturer’s instructions. ROS generation levels were detected using a fluorescence-labelled probe DCFH-DA (Beyotime Biotech). Further analysis was performed on a three-laser FACSscan (Cytek Development, Fremont, CA, United States), and all experiment data were processed with Flow Jo software (version 10.4.0, TreeStar, Inc.).

### Capillary-Based Western Blot

The total protein lysates of kidney tissues and HK2 cells were extracted with the T-PER tissue protein extraction buffer and M-PER mammalian protein extraction buffer (Pierce Biotechnology, Thermo Scientific, United States), containing 1% protease inhibitor cocktail (B14001; Bimake) and 1% phosphatase inhibitor (B15001; Bimake), respectively. For protein expression detection, we used an automated capillary western blot (Jess, Protein Simple; San Jose, CA, United States), which is considered as more rapid, efficient compared to traditional western and has also been proved in numerous studies (Nanki et al., 2018; Diebold et al., 2019). All experimental procedures were strictly carried out following the manufacturer’s instructions. Chemiluminescence data were produced by the Compass for SW software (version 3.8 for Mac OS X, Protein Simple). All values were normalized to GAPDH.

### Statistical Analysis

All experimental data are presented as mean±SD. The unpaired two-tailed Student’s *t*-test was used for two group comparisons and one-way ANOVA followed by Tukey’s multiple comparison test for multiple group comparisons. Statistical significance was considered as *p*<0.05. GraphPad Prism 8.0 (La Jolla, CA, United States) was used for statistical analysis.

## Results

### CHR Ameliorates the Damage of Kidney Histology and Function in CDDP-AKI Mice Model

The *in vivo* effect of CHR on AKI induced by CDDP was firstly evaluated by a CDDP-induced kidney injury mouse model set up by a single intraperitoneal CDDP injection. The overall experimental design was depicted as Figure 1B. As shown in Figure 1C, CDDP induced a large area of cell death in the tubular epithelium in the cortex, as well as swelling, featured fragmentation, and a few casts, which were relieved by pre-treatment with CHR. Similarly, compared with the control group, the tubular injury score in the CDDP+vehicle group was significantly higher (*p*<0.0001, Figure 1D). Pre-treatment with CHR partly rescued the damage induced by CDDP (*p*<0.0001, Figure 1D). Considering that different doses of CHR might induce different response, the dose effect of CHR on tubular injury score was analyzed. Pre-treating with 20 or 40mg/kg CHR had no difference in tubular injury scores (*p*>0.05, Figure 1D).

**Figure 2:**
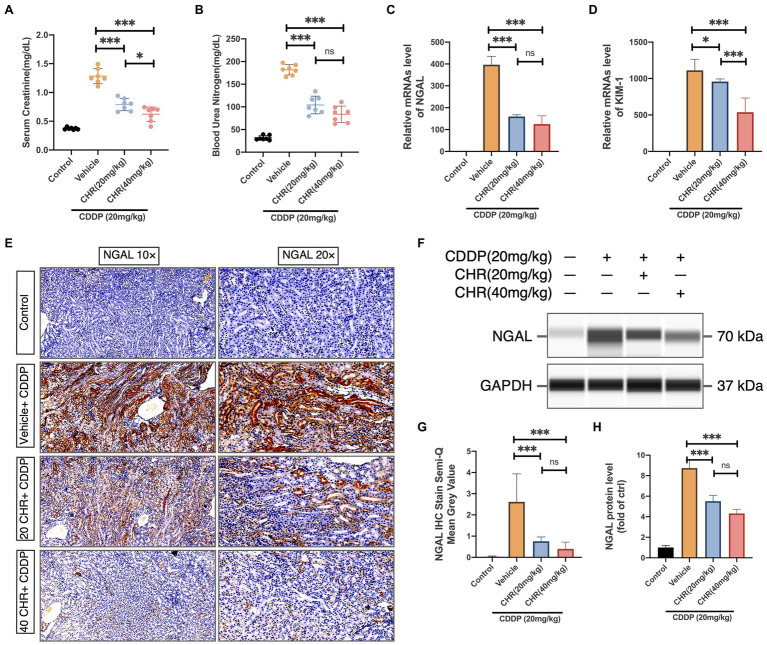
Chrysophanol pretreatment attenuated CDDP-induced kidney dysfunction of C57 mice. **(A)** Serum Creatinine (SCr) levels. **(B)** Blood urea nitrogen (BUN) levels. Results of SCr and BUN levels show that pretreatment of CHR protected kidney function in CDDP-acute kidney injury (AKI) **(C)** Neutrophil gelatinase-associated lipocalin (NGAL) mRNA expression. **(D)** Kidney injury molecule 1 (Kim-1) mRNA expression. NGAL and Kim-1 are classical kidney injury markers. **(E)** The representative graphs of immunochemistry (IHC) with NGAL antibody are presented. **(F)** The expression of NGAL and GAPDH protein detected by capillary blot. **(G)** Semi-quantification and statistical analysis of NGAL expression for IHC staining in kidney tissues. **(H)** Quantitative and statistical analysis of relative NGAL level normalized to GAPDH in capillary blot. ^*^p<0.05 and ^***^p<0.005. ns, no statistical difference.

The effects of CHR on renal function were analyzed by detecting the level of serum creatinine (Scr) and BUN (Figures 2A–B). Compared to the Vehicle+CDDP group, CHR pretreatment partly recovered the renal function and significantly decreased the Scr (20mg/kg CHR+CDDP vs. vehicle+CDDP: 1.28±0.05 vs. 0.79±0.04, *p*<0.0001; 40mg/kg CHR+CDDP vs. vehicle+CDDP: 1.28±0.05 vs. 0.63±0.05, *p*<0.0001; Figure 2A) and BUN level (20mg/kg CHR+CDDP vs. vehicle+CDDP: 181.8±4.28 vs. 104.2±7.35, *p*<0.0001; 40mg/kg CHR+CDDP vs. vehicle+CDDP: 181.8±4.28 vs. 83.69±6.84, *p*<0.0001; Figure 2B).

To further evaluate the effects of CHR on CDDP-AKI, the expression levels of two proximal tubular damage markers, NGAL and kidney injury molecule 1 (KIM-1), were also detected. CDDP elevated the expression of NGAL and KIM-1, which was attenuated by CHR (Figures 2C–H).

### CHR Attenuated NOX Mediated Oxidative Stress Induced by CDDP in the Mouse Kidney

**Figure 3:**
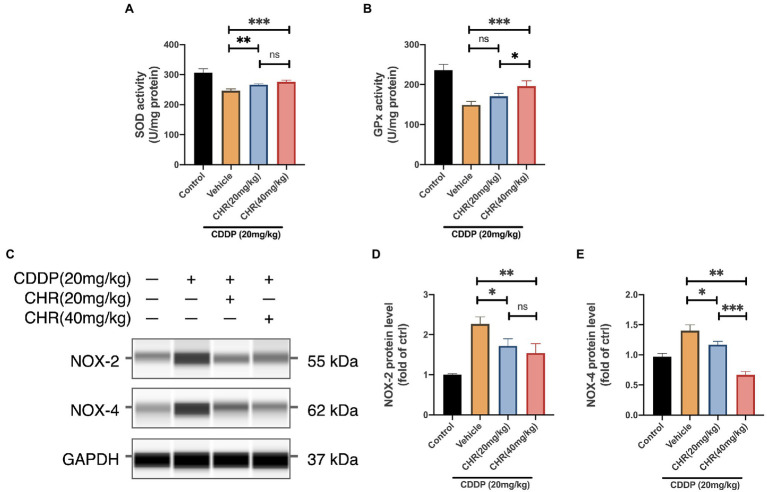
Chrysophanol pretreatment relieved CDDP-induced tissue oxidative damage of C57 mice *via* NOX signaling inhibition. **(A)** The serum superoxide dismutase (SOD) enzyme activity. **(B)** The serum glutathione peroxidase (GPx) enzyme activity. **(C)** The protein expression of NOX-2, NOX-4, and GAPDH detected by capillary blot. **(D)** Quantitative and statistical analysis of relative NOX-2 level in kidneys. **(E)** Quantitative and statistical analysis of relative NOX-4 level in kidneys. ^*^p<0.05, ^**^p<0.01, and ^***^p<0.005. ns, no statistical difference.

More and more studies support that the occurrence and development of acute and CKD are closely related to the imbalance of oxidative stress. Besides, CHR has also been proven to have antioxidant properties. Therefore, the activity of SOD and GPx, which were antioxidant response elements, was measured. CDDP-AKI mice which received 20 or 40mg/kg CHR had significantly decreased SOD activities compared to the vehicle group (*p*<0.05, Figure 3A). Consistently, 40mg/kg CHR pretreatment significantly reduced the GPx activity (*p*<0.01, Figure 3B). Compared to the vehicle group, the GPx activity of the 20mg/kg CHR pretreatment group was decreased, however, not statistically meaningful (*p*=0.0858, Figure 3B). Moreover, the protein levels of NOX2 and NOX4 were verified. The results suggested that lower expression levels of NOX2 and NOX4 were both observed in the CDDP-AKI mice pretreated with 20mg/kg CHR and 40mg/kg CHR (*p*<0.05, *p*<0.01, respectively; Figures 3C–E). In addition, the NOX4 protein expression in CHR treatment groups was declined in a dose-dependent manner as shown in Figure 3E. These results indicated that CHR could attenuate oxidative stress induced by CDDP through the inhibition of NOX enzyme activation.

### CHR Attenuated CDDP-Induced p53-Dependent Apoptosis

**Figure 4:**
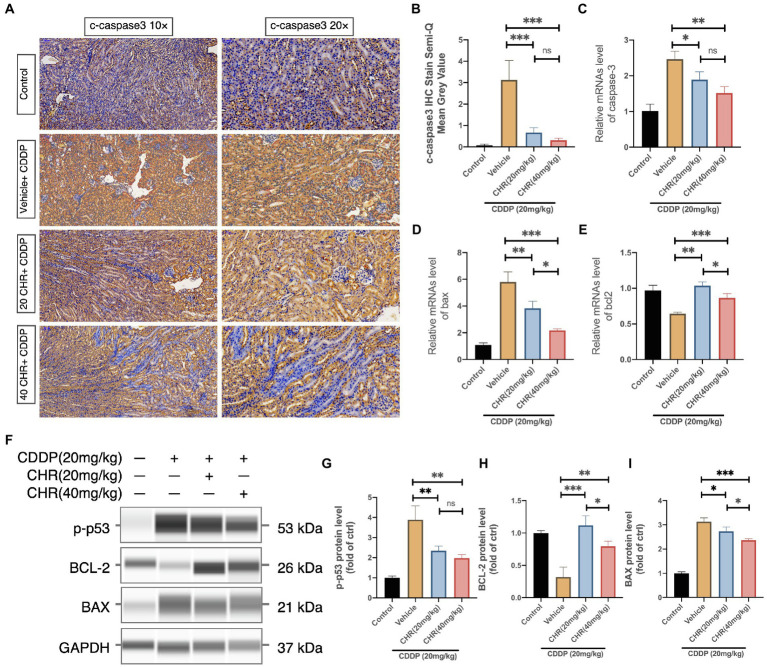
Chrysophanol pretreatment relieved CDDP-induced nephritic apoptosis of C57 mice *via* inhibition of p53 activation. **(A)** Representative graphs of IHC with c-caspase 3 antibody. **(B)** Semi-quantification and statistical analysis of c-caspase 3 expression for IHC staining in kidney tissues. **(C)** Caspase 3 mRNA expression. **(D)** Bax mRNA expression. **(E)** Bcl-2 mRNA expression. **(F)** The protein expression of p-p53, BCL-2, BAX, and GAPDH detected by capillary blot. **(G)** Quantitative and statistical analysis of relative p-p53 level in kidneys. **(H)** Quantitative and statistical analysis of relative BCL-2 level in kidneys. **(I)** Quantitative and statistical analysis of relative BAX level in kidneys. ^*^p<0.05, ^**^p<0.01, and ^***^p<0.005. ns, no statistical difference.

P53-dependent apoptosis could be activated by oxidative stress. Hence, several biomarkers related to p53-dependent apoptosis were detected. Firstly, we found that CHR could attenuated the cleavage and expression of caspase 3 accelerated by CDDP administration (Figures 4A–C). In addition, CHR pre-treatment could inhibit the mRNA and protein levels of Bax and increase the expression of Bcl2 (Figures 4D–I). Furthermore, we detected the protein level of phosphorylated p53 (ser15). As shown in Figures 4F–G, compared with the control group, the phosphorylated p53 was significantly increased, after treating with CDDP. Besides, both low and high dose CHR pretreatment could suppress its phosphorylation induced by CDDP. These data suggested that CHR inhibited CDDP-induced p53-dependent apoptosis *in vivo*.

### CHR Pretreatment Inhibited CDDP-Induced Inflammation Through NF-κB Signal Pathway.

**Figure 5:**
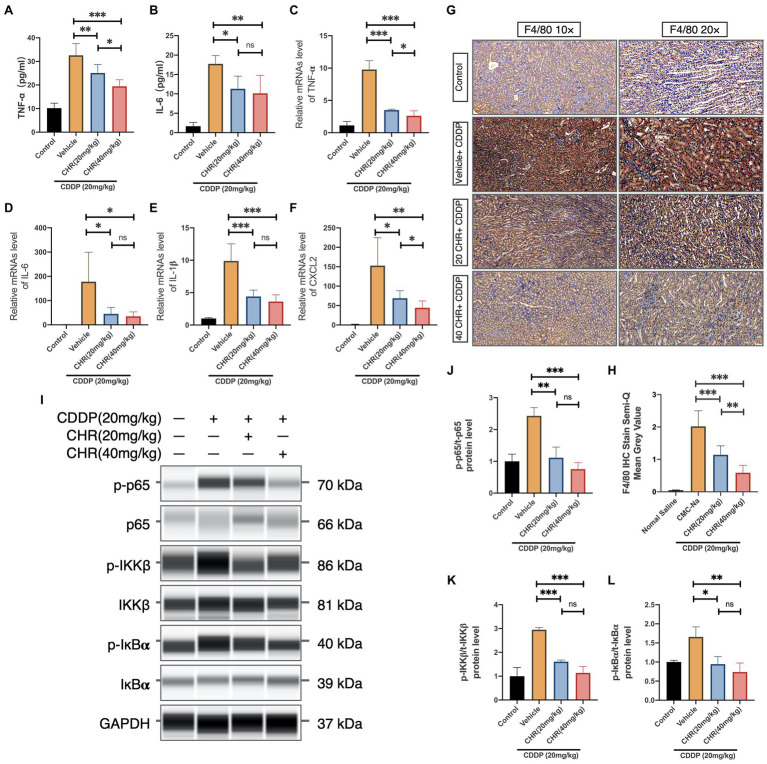
Chrysophanol pretreatment relieved CDDP-induced inflammation of C57 mice *via* inhibition of IKK/IκBα/p65 NF-κB signaling. **(A)** The tumor necrosis factor alpha (TNF-α) serum level. **(B)** The interleukin-6 (IL-6) serum level. **(C)** TNF-α mRNA expression in mouse kidney tissue. **(D)** IL-6 mRNA expression in mouse kidney tissue. **(E)** Interleukin 1 beta (IL-1β) mRNA expression in mouse kidney tissue. **(F)** Chemokine ligand 2 (CXCL2) mRNA expression in mouse kidney tissue. **(G)** Kidney tissues were subjected to IHC staining with antibody against F4/80. **(H)** Semi-quantification and statistical analysis of F4/80 expression for IHC staining in kidney tissues. **(I)** The protein expression of p-p65, p65, p-IKKβ, IKKβ, p-IκBα, IκBα, and GAPDH detected by capillary blot. **(J)** Quantification and statistical analysis of the expression of p-p65 normalized by total p65. **(K)** Quantification and statistical analysis of the expression of p-IKKβ normalized by total IKKβ. **(L)** Quantification and statistical analysis of the expression of p-IκBα normalized by total IκBα. ^*^p<0.05, ^**^p<0.01, and ^***^p<0.005. ns, no statistical difference.

To evaluate whether CHR was capable of attenuating CDDP-induced tissue inflammation, the serum inflammatory cytokine level was firstly measured at 72h after CDDP stimulation. As expected, serum TNF-α and IL-6 levels were attenuated in both low and high CHR pretreatment groups, compared to vehicle group (Figures 5A,B). What is more, CHR administration could block the increasing mRNA expression of TNF-α, IL-1β, IL-6, and Chemokine ligand 2 (CXCL2) induced by CDDP (Figures 5C–F). The degree of macrophage infiltration in mouse kidney was also detected by staining F4/80, which is a macrophage marker. In CDDP-induced groups, F4/80 positive cells were observed in CDDP-AKI mouse model. CHR pretreatment significantly decreased the degree of macrophage infiltration (Figures 5G–H). NF-κB activation played an important role in inflammation,

Thus, the effects of CHR on protein expression of phospho-IκBα, total IκBα, phospho-IKK, total IKK, phospho-NF-κB p65, and total NF-κB p65 subunit were evaluated (Figure 5I). We found that CDDP injection increased phosphorylation p65 IKKβ and IκBα, which were partly blocked by CHR pre-treatment (Figures 5J–L). To conclude, these results indicated that CHR was able to partially protect mouse kidneys against inflammatory insult caused by CDDP, presumably through suppressing canonical NF-κB signaling pathway.

### CHR Alleviated CDDP-Induced Cell Viability Decrease in HK-2 Cell Line

**Figure 6:**
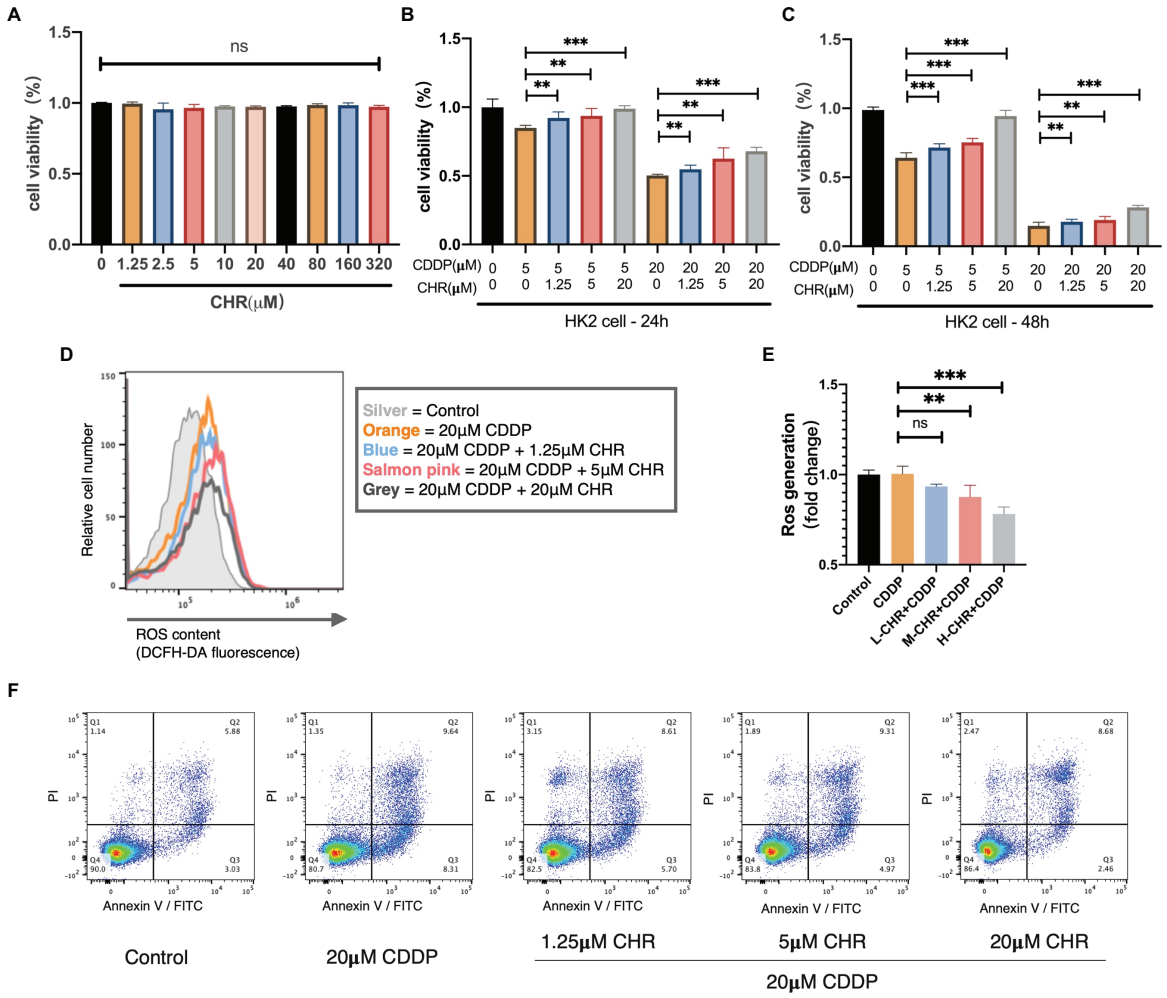
Chrysophanol attenuated CDDP-caused nephrotoxicity *in vitro*. **(A)** Effect of different doses of CHR on viability of HK2 cells. HK2 cells were cultured with CHR at different doses (1.25, 2.5, 5,10, 20, 40, 80, 160, and 320μmol/L) for 48h, and the cell viability were measured by CCK8 assay. **(B–C)** CHR restored cell viability in CDDP-treated HK2 cells. HK2 cells were pre-incubated with the different doses of CHR (1.25, 5, and 20μmol/l) for 24h before stimulation with CDDP (5 and 20μmol/L). **(D)** Cellular reactive oxygen species (ROS) levels were measured using DCFH-DA probe and detected by flow cytometry. HK2 cells were cultured with low concentration (1.25μmol/L), medium concentration (5μmol/L), and high concentration (20μmol/L) CHR for 24h. And then stimulated with 20μmol/L CDDP for 16h and detected. **(E)** Quantification of intracellular ROS generation under different conditions. **(F)** Cell apoptosis percentage determined by flow cytometry, staining using Annexin-V/PI. ^**^p<0.01, and ^***^p<0.005. ns, no statistical difference.

Furthermore, we explored the inhibition effect of CHR on kidney damage induced by CDDP in human kidney tubule epithelial HK2 cell line. Firstly, we investigated the cytotoxicity of CHR. As shown in Figure 6A, the results indicated that it did not show any sign of toxicity even at high concentrations (320μmol/L). Then, HK2 cells were incubated with CHR at different doses (0, 1.25, 5, and 20μmol/L) for 24h before stimulation with CDDP (5 or 20μmol/L). CCK8 assay results revealed that CHR significantly reversed induced viability reduction in a dose-dependent manner, suggesting that it could alleviate CDDP-caused cell viability decrease in HK-2 cells (Figures 6B–C).

### CHR Impaired ROS Generation and Apoptosis Induced by CDDP in HK-2 Cell Line

To further excavate how CHR protect against CDDP-caused tubular cytotoxicity, we detected the change of ROS generation. As Figures 6D,E show, ROS production stimulated by CDDP could be partially alleviated by CHR treatment. Moreover, the protective effect against ROS generation of CHR was more significant with the higher dose. After that, the effect of CHR on cell apoptosis was detected. As the dose of CHR increases, its protective effect against apoptosis becomes more obviously. In conclusion, CHR reduced CDDP-induced HK2 cellular ROS generation and apoptosis in a dose-dependent manner.

## Discussion

As one of the most widely prescribed platinum drugs, CDDP is frequently used in treating various cancers. However, there is no effective treatment to overcome its common side effects. Currently, several antioxidants, such as capsaicin, curcumin, ellagic acid, lycopene, and resveratrol, were identified as nephroprotective drugs against CDDP-AKI (Gómez-Sierra et al., 2018). However, none of these proved themselves in clinical trials and it is still urgent to seek novel agents or targets to overcome CDDP-AKI.

Chrysophanol is one natural compound isolated from the rhizome of a Chinese traditional drug Rheum palmatum L. This herb is frequently used in ancient China for treatment of stomach ailments, dropsy, and fevers, or as a “cathartic” which means constipation relieving. After the first International Symposium on Rhubarb held in China in 1990, a growing body of research focused on evaluating this medicine and analyzed the mechanism of it. CHR was found to be a major agent in the methanolic extracts of R. palmatum L. Animal and cellular research have made substantial proof in its benefit to lung cancer, oral cancer, breast cancer, and many other tumors, as well as cardiovascular diseases, cerebral ischemia/reperfusion injury, and ethanol-induced liver injury. Mechanically, it exerts anticancer effects through cancer cell proliferation inhibition *via* EGFR/mTOR signaling pathway, through cell death induction *via* calcium-dependent ROS production or through epithelial-mesenchymal transition (EMT) suppression *via* blocking the HIF-1α activation of PI3k/Akt signaling pathway (Lee et al., 2011; Ni et al., 2014; Deng et al., 2019). Moreover, we found that CHR effectively protects against CDDP-AKI here in this study. For the research on AKI induced by CDDP, a typical anticancer drug, it is of great significance to seek agents such as CHR that may possess anti-tumor potential and kidney protective activity at the same time.

To date, much of the work has been focused on the cardiovascular-protective, neuroprotective and hepaprotective role of CHR (Prateeksha and Singh, 2019; Xie et al., 2019; Su et al., 2020). *In vivo* animal conditions implicated that CHR attenuated high fat-induced cardiac injury (through nrf2 dependent antioxidant, antiinflammation), cerebral ischemia/reperfusion injury (through suppression of the NALP3 inflammasome), LPS/D-GalN-caused hepatic injury (through RIP140/NF-κB signaling inhibition), and DOX-induced cardiotoxicity (through antiapoptotic regulation), etc. (Zhang et al., 2014; Jiang et al., 2016; Lian et al., 2017; Lu et al., 2019). Apart from its organ protective potentiality, the in-body process characteristics also caught our attention. By a rat pharmacokinetic study, CHR exhibited high lipophilicity and underwent rapidly absorption and distribution after orally administrated. Concentration determination also indicated that it was eliminated by kidney excretion instead of liver metabolism (Chen et al., 2014). Such potential organ protective potential together with the relatively high drug concentration in the kidney may set the stage for the protection of injured kidney. Previous studies in the field of nephrology have mostly described its anti-fibrosis properties *via* TGF-β inactivation in CKD and DN (Dou et al., 2020; Guo et al., 2020). Thus the possible roles of CHR on AKI had not been assessed. Here in this study, an overall protective role of CHR in CDDP-AKI was confirmed by evidence collected both *in vivo* and *in vitro*: CHR administration alleviated kidney function and morphology loss in mice injured by CDDP injection. Furthermore, CHR significantly suppressed ROS production in injured tubular epithelial cells. We propose that CHR, an effective ingredient of traditional Chinese medicine, is a good candidate to protect kidney tubular cells from cytotoxic insults, with significant therapeutic potential for the treatment of CDDP-AKI.

*In vivo*, we applied an intraperitoneal injection of CDDP to induce CDDP-AKI mouse model. The kidney tissue damage and functional alterations caused by CDDP could be significantly relieved by pretreatment of CHR. In this study, we found that the elevation in CREA and BUN, which are classic indicators of acute kidney damage and dysfunction, was attenuated in 20 and 40mg/kg CHR treated groups. Due to that NGAL and Kim-1 are expressed at a very low level in normal tissues, while they will be markedly induced in injured epithelial cells; they can serve as promising AKI biomarkers (Nguyen and Devarajan, 2008; Peralta et al., 2012; Fan et al., 2018). We also detected the influence of CHR on the expression of NGAL and Kim-1. The results indicated that CHR pretreatment inhibited the expression of NGAL and Kim-1 induced by CDDP injection. In addition, CHR pre-incubation rescued the cell viability in a dose-independent manner. Taken together, these findings support that CHR might be a useful nephroprotective pharmaceutical very well.

In the occurrence and development of CDDP-AKI, ROS and oxidative stress are subsequently involved (Tomsa et al., 2019). Under normal physiologic condition, antioxidant enzymes including SOD and GPx are at the first lines to protect cell against oxidative stress *via* lipid peroxidative prevention. The imbalance between endogenous ROS production and antioxidant activity, which is considered as oxidative stress, occurs under pathological circumstances. We demonstrated that CHR had the protective potential against cellular ROS generation activated by CDDP *in vitro*. Consistently, *in vivo* study showed that both SOD and GPx activity exhausted by 20mg/kg CDDP were slightly increased after the application of CHR in the kidney tissues. These results together indicated the antioxidant ability of CHR during the development of CDDP-AKI. Then, we analyzed the antioxidant mechanism and noticed that NOX2 and NOX4 were significantly downregulated in the CHR pretreatment mouse groups. Under pathological circumstances with CDDP accumulation, excessive ROS mainly generated by NOX may exceed the normal antioxidant ability to neutralize them, causing oxidative stress and aberrant signal transduction. Our data showed that both 20 and 40mg/kg CHR pretreatment significantly inhibited the protein expression of NOX2 and NOX4, indicating that CHR may attenuate CDDP-AKI by suppressing NOX-mediated oxidative stress.

Previous studies have shown the involvement of p53 in CDDP nephrotoxicity (Pabla and Dong, 2008). Notably, p53 knockout mice displayed ameliorated kidney damage and tubular cell apoptosis in CDDP-AKI. Analogously, pifithrin-a, a p53 inhibitor, could ameliorate CDDP-AKI in WT animals (Wei et al., 2007). Mechanically, after oxidative stress occurs, p53 is immediately activated, which is characterized by rapid accumulation of p53 and subsequent phosphorylation in stressed cells. Phosphorylated p53 may cause mitochondrial dysfunction and subsequent caspase cascade activation *via* the directly activation of pro-apoptotic BCL-2 family members, leading to cell apoptosis (Jiang et al., 2004). Thus, we focused on the expression of phosphorylated p53, proapoptotic BAX, antiapoptotic BCL-2, and cleaved-caspase 3. The increased p-p53 and BAX together with decreased BCL-2 in kidneys of CDDP-AKI mice were significantly rescued by CHR. Moreover, the expression of cleaved-caspase 3 also reflected its anti-apoptotic effect. *In vitro*, the exposure to 20μM CDDP induced HK2 cell apoptosis, which could be rescued by CHR pre-incubation. Hence, CHR may ameliorate CDDP-induced tubular cell apoptosis *via* p53-regulated BAX/BCL-2 axis.

It is noteworthy that oxidative stress can also activate the canonical NF-κB pathway, thereby increasing the expression of pre-inflammatory genes including TNF-α, IL-1β, and IL-6, which consequently cause continuous inflammation and tissue injury (Siomek, 2012; Weifeng et al., 2016; Lingappan, 2018). In the current study, CDDP significantly increased TNF-α, IL-1β, and IL-6 levels in injured mouse kidney. The percent of F4/80-positive macrophages was also increased after CDDP injection. It is noteworthy that these changes could all be rescued by CHR. Our results indicated that CHR had the ability of reducing CDDP-caused kidney inflammatory responses, since it has been shown that the phosphorylation of IKK, IκBα, and p65 subunit is the crucial upstream event of NF-κB activation. We next evaluated the effect of CHR on these molecules by capillary WB. We revealed that CHR suppressed IKK/IκBα/p65 NF-κB signaling, thereby alleviating the inflammatory response caused by the activation of this signal.

The main limitation of this study is the gap between nephrology and oncology. Bridging this gap is crucial as only patients with cancer receive CDDP treatment. Therefore, the next step of our research will involve the incorporation of cancer into a clinically relevant model of CDDP treatment (Huang et al., 2019). We are planning a future study to use the model of xenograft nude mice bearing carcinoma cells and treated with CDDP with/without CHR, respectively. Since CHR possesses significant anticancer activity as mentioned above, we believe CHR can be a good candidate to improve CDDP-induced anticancer activity and to prevent CDDP-AKI at the same time.

**Figure 7:**
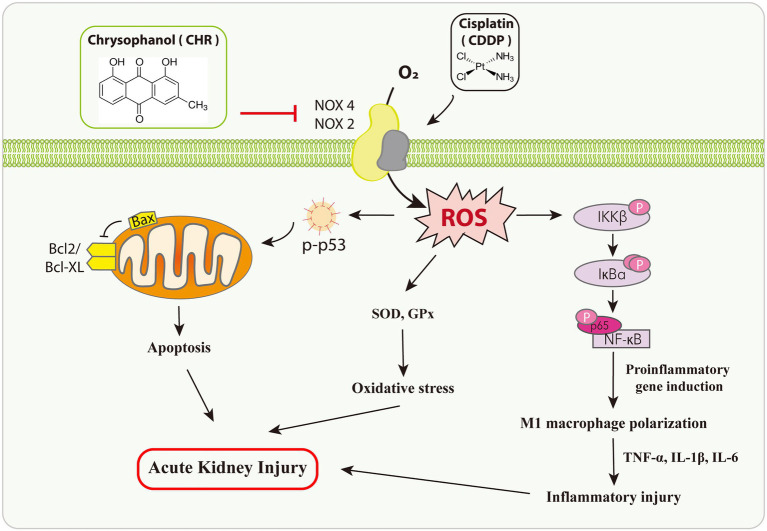
Diagram presents the therapeutic potential of CHR on CDDP-induced AKI.

Collectively, in this study, we observed pretreatment CHR ameliorated CDDP-AKI both *in vivo* and *in vitro* (Figure 7). While we could not exclude the involvement of other targets, our research suggests that the protective effect of CHR on CDDP-AKI is mediated, at least in part, through suppressing NOX mediated oxidative stress and the subsequent p53-dependent apoptosis as well as NF-κB inflammation signaling pathway. The current findings might have good implications regarding the development of therapeutic approaches against CDDP-AKI.

## Data Availability Statement

The original contributions presented in the study are included in the article/Supplementary Material, further inquiries can be directed to the corresponding authors.

## Ethics Statement

The animal study was reviewed and approved by the Institutional Animal Care and Use Committee of Central South University.

## Author Contributions

SM and WZ provided conception. SM, HX, WH, YG, and HZ carried out experiments and formal data analysis. SM, XL, and WZ drafted, edited, and revised the manuscript. All authors contributed to the article and approved the submitted version.

## Funding

This study was funded by the National Natural Science Foundation of China (No.81874329 and No.82073945), Scientific Research Project of Hunan Provincial Health Commission (B2013-097), Science and Technology Innovation Program of Hunan Province (2018SK50907), and the Fundamental Research Funds for Central Universities of Central South University (No. 2019zzts097).

## Conflict of Interest

The authors declare that the research was conducted in the absence of any commercial or financial relationships that could be construed as a potential conflict of interest.

## Publisher’s Note

All claims expressed in this article are solely those of the authors and do not necessarily represent those of their affiliated organizations, or those of the publisher, the editors and the reviewers. Any product that may be evaluated in this article, or claim that may be made by its manufacturer, is not guaranteed or endorsed by the publisher.
