# Corrigendum: Chrysophanol Relieves Cisplatin-Induced Nephrotoxicity *via* Concomitant Inhibition of Oxidative Stress, Apoptosis, and Inflammation

**DOI:** 10.3389/fphys.2021.794302

**Published:** 2021-12-09

**Authors:** Siqing Ma, Heng Xu, Weihua Huang, Yongchao Gao, Honghao Zhou, Xiong Li, Wei Zhang

**Affiliations:** ^1^Department of Clinical Pharmacology, Xiangya Hospital, Central South University, Changsha, China; ^2^Hunan Key Laboratory of Pharmacogenetics, Institute of Clinical Pharmacology, Central South University, Changsha, China; ^3^Engineering Research Center of Applied Technology of Pharmacogenomics, Ministry of Education, Changsha, China; ^4^National Clinical Research Center for Geriatric Disorders, Changsha, China; ^5^Department of Laboratory Medicine, State Key Laboratory of Biotherapy and Cancer Center, West China Hospital, Sichuan University, Chengdu, China; ^6^The First Affiliated Hospital of Guangdong Pharmaceutical University, Guangdong, China

**Keywords:** chrysophanol, cisplatin, acute kidney injury, oxidative stress, apoptosis, inflammation

**Error in Figure**

In the original article, there was a mistake in Figure 4 as published. The wrong figure was published. The corrected capillary blot of BAX and GAPDH in Figure 4F is now consistent with the original data submitted during initiation validation process. The corrected Figure 4 appears below.

**Figure 4:**
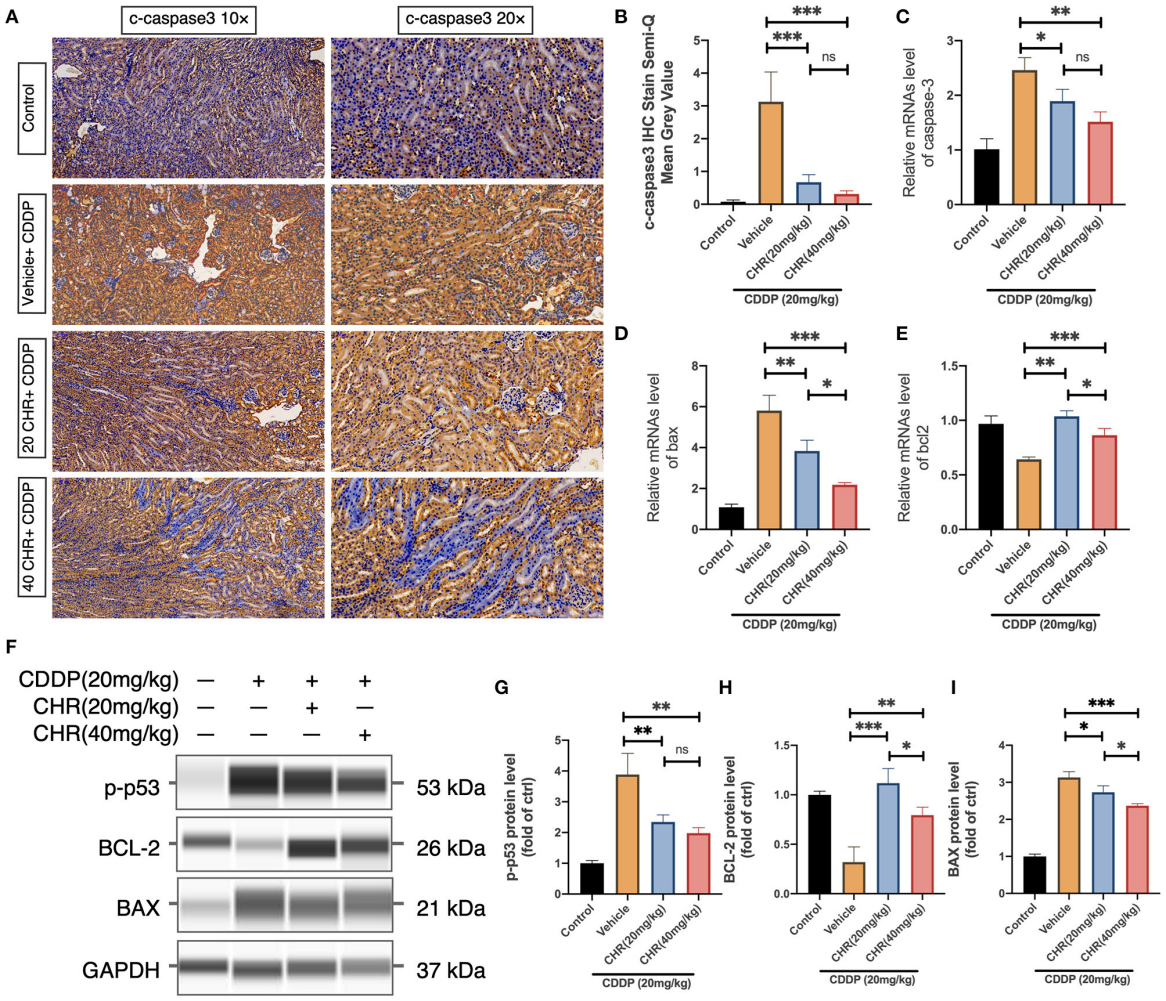
Chrysophanol pretreatment relieved CDDP-induced nephritic apoptosis of C57 mice via inhibition of p53 activation. **(A)** Representative graphs of IHC with c-caspase 3 antibody. **(B)** Semi-quantification and statistical analysis of c-caspase 3 expression for IHC staining in kidney tissues. **(C)** Caspase 3 mRNA expression. **(D)** Bax mRNA expression. **(E)** Bcl-2 mRNA expression. **(F)** The protein expression of p-p53, BCL-2, BAX, and GAPDH detected by capillary blot. **(G)** Quantitative and statistical analysis of relative p-p53 level in kidneys. **(H)** Quantitative and statistical analysis of relative BCL-2 level in kidneys. **(I)** Quantitative and statistical analysis of relative BAX level in kidneys. *p < 0.05, **p < 0.01, and ***p < 0.005. ns, no statistical difference.

The authors apologize for this error and state that this does not change the scientific conclusions of the article in any way. The original article has been updated.

## Publisher's Note

All claims expressed in this article are solely those of the authors and do not necessarily represent those of their affiliated organizations, or those of the publisher, the editors and the reviewers. Any product that may be evaluated in this article, or claim that may be made by its manufacturer, is not guaranteed or endorsed by the publisher.

