# Supplementary material for: Chrysophanol Relieves Cisplatin-Induced Nephrotoxicity via Concomitant Inhibition of Oxidative Stress, Apoptosis, and Inflammation
Source: Front Physiol. 2021 Sep 29;12:706359. doi: 10.3389/fphys.2021.706359 (PMC8514135; doi:10.3389/fphys.2021.706359)
Supplement: Supplementary file 1 [file Table_1.DOCX]

Supplement Table 1. Primer sequences used for RT-qPCR assays.

| Gene |  | Sequences | NCBI GeneID |
| --- | --- | --- | --- |
| NGAL | F | GCAGGTGGTACGTTGTGGG | 16819 |
|  | R | CTCTTGTAGCTCATAGATGGTGC |  |
| KIM-1 | F | ACATATCGTGGAATCACAACGAC | 171283 |
|  | R | ACAAGCAGAAGATGGGCATTG |  |
| CASPASE-3 | F | TGGTGATGAAGGGGTCATTTATG | 12367 |
|  | R | TTCGGCTTTCCAGTCAGACTC |  |
| BAX | F | TGAAGACAGGGGCCTTTTTG | 12028 |
|  | R | AATTCGCCGGAGACACTCG |  |
| BCL2 | F | GAGAGCGTCAACAGGGAGATG | 12043 |
|  | R | CCAGCCTCCGTTATCCTGGA |  |
| TNF-𝛂 | F | CAGGCGGTGCCTATGTCTC | 21926 |
|  | R | CGATCACCCCGAAGTTCAGTAG |  |
| IL-6 | F | TCTATACCACTTCACAAGTCGGA | 16193 |
|  | R | GAATTGCCATTGCACAACTCTTT |  |
| IL-1𝛃 | F | GCAACTGTTCCTGAACTCAACT | 16176 |
|  | R | ATCTTTTGGGGTCCGTCAACT |  |
| CXCL2 | F | CCAACCACCAGGCTACAGG | 20310 |
|  | R | GCGTCACACTCAAGCTCTG |  |
| GAPDH | F | AGGTCGGTGTGAACGGATTTG | 14433 |
|  | R | TGTAGACCATGTAGTTGAGGTCA |  |

F: Forward; R: Reverse; NGAL: Neutrophil gelatinase-associated lipocalin; KIM-1: Kidney Injury Molecule-1; BAX: BCL2 Associated X; BCL2: B-cell lymphoma 2; TNF-𝛂: Tumor necrosis factor alpha; IL-6: Interleukin 6; IL-1𝛃: Interleukin 1 beta; CXCL2: Chemokine (C-X-C motif) ligand 2; GADPH: Glyceraldehyde 3-phosphate dehydrogenase
